# Dataset on the production of predominantly male tilapia progeny using two malawian tilapias, *Oreochromis karongae* and *Oreochromis shiranus*

**DOI:** 10.1016/j.dib.2020.105716

**Published:** 2020-05-18

**Authors:** Alfred Maluwa, Madalitso Snake, Hastings Zidana, Petros Chigwechokha, Moses Simwaka

**Affiliations:** a Directorate of Research, Malawi University of Science and Technology, Malawi; b Department of Fisheries and Aquatic Sciences, Mzuzu University, Malawi; c National Aquaculture Centre, Domasi, Malawi

**Keywords:** All male tilapia, Tilapia interspecific hybridization, Tilapia hormonal sex reversal, Sex determination system in Malawian tilapias, Oreochromis shiranus, Oreochromis karongae, Malawian farmed tilapias, Aceto-carmine juvenile sexing method

## Abstract

A dataset is presented of an experiment that was conducted to compare the proportions of males obtained from hormonal sex reversed pure strain of *Oreochromis shiranus* and from interspecific hybridization of *O.karongae* male x *O. shiranus* female. Part of the data in the dataset were published in a journal article https://doi.org/10.1016/j.aqrep.2020.100274. The data were generated from four SETs of treatments of an experiment that was conducted at the National Aquaculture Center, in Malawi. The first SET of treatment comprised hybrids from interspecific crossing of *O.karongae* male and *O. shiranus* female in a pond based breeding hapa 1. SETs 2 and 4 were for fry from hapa spawned pure cross of *O.shiranus* males and females in a pond based breeding hapa 2. SET 3 comprised fry from pure cross of *O. shiranus* males and females under controlled temperature (27°C) in an indoor re-circulatory hatchery (Tables 1 to 4). During the first part of the experiment, the fry was raised for 28 days in tanks at three replicates and was fed a fry formulated feed containing 38% crude protein, three times a day. However, for SETs 2 and 3, the feed contained 17α-methyl testosterone at 60mg/kg of feed. The second part of the experiment involved growth performance testing of fry in each SET. The growth experiment was conducted in rearing hapas that were inserted in a common pond for a period of 70 days. The body weight data were collected every 14 days (Tables 1 to 4). On the 70th day, the proportions of males and females in the four SETs was determined using Aceto-carmine staining method (Table 5). The pictures of the stained male and female gonads under compound microscope are presented in the published journal article at https://doi.org/10.1016/j.aqrep.2020.100274. Dataset presented in this paper is for body weights of fish from day 0 to day 70 and proportion of males and females in each SET on the 70^th^ day of the experiment (Tables 1, 2, 3, 4 and 5). The data were analyzed using SPSS version 20.0. Chi-square goodness of fit test was used to investigate if the observed sex ratios significantly deviated from the expected sex ratios at 5% level of significance. The differences among fish body weights were determined using Analysis of Variance and significantly different means were separated using Duncan's multiple range test at the 5% test level of significance. These data are useful for various stakeholders that are interested in sexing juvenile tilapia and for scientists that conduct tilapia sex reversal experiments. This data can also guide hatchery managers during commercial production of all male tilapias which grow faster than those in the mixed sex tilapia culture.

Specifications table

**Table:** 

Subject	Agricultural and Biological Sciences
Specific subject area	Aquaculture or Aquatic Sciences
Type of data	Tables
How data were acquired	Data on body weighs were generated from physically weighing fish and recording the body weights every 14 days for 70 days to the nearest 0.1g using an Octadem Weighing Scale (model WBW 1.5m 1500g x:0.59). Photographs of Aceto-carmine stained fish (male and female) gonads were taken using a camera that was mounted on a microscope (Stereoscopic microscope Model H-III). Observations of gonads was done with a compound microscope model XSZ-107T at 100x and 400 x magnifications. The pictures of the stained gonads are available in the main journal article at https://doi.org/10.1016/j.aqrep.2020/100274. For the sex ratios, the data were generated by sexing the fish on the 70^th^ day of the experiment and the sex ratios were obtained from the number of males and females in each replicate within a SET.
Data format	Raw data are presented as a dataset in MS word formatted tables
Parameters for data collection	Body weight data were collected through taking fish body weight using weighing scale to 0.1g every 14 days for 70 days. The proportion of males was generated from the number of males and females in the interspecific hybridizations and pure cross hormone reversed fish treatments and from the control groups.
Description of data collection	Fertilized eggs from interspecific hybridization of *O. karongae* male x *O. shiranus* female and pure crosses of *O. shiranus x O. shiranus* were artificially incubated at 27°C in the hatchery. After hatching and at swim up, the pure strains were fed a hormone incorporated feed with a sex reversal hormone (17 α-methyl testosterone) at 60 mg/kg of feed for 28 days. Body weight was recorded on all fish every 14 days for 70 days. The fish gonads were examined after staining with aceto-carmine solution after 70 days of rearing and were observed under a compound microscope at 100 x and 400x magnifications. The number of males and females was physically counted from each replicate within a SET.
Data source location	The experiment was conducted at the National Aquaculture Centre, Domasi, Zomba, Southern Region of Malawi in Malawi Latitude: -15,3085, Longitude: 35,3932 111 N -15 18’31’’, E 3523’36”
Data accessibility	Data are incorporated within this article
Related research article	Snake, M., Maluwa, A., Zidana, H., Chigwechokha, P., Simwaka, M. (2020). Aquaculture Reports. https://doi.org/10.1016/j.aqrep.2020.100274.

## Value of the data

• These data are useful to guide researchers in aquaculture and fish breeding as they provide details of the methodology and type of data to be collected during similar studies
• Researchers in fish biology, fish breeding and genetics, aquatic sciences and aquaculture would benefit from this data
• The data would assist in the design of experiments, broodstock selection, deciding on sampling schedule and statistical analysis of similar datasets
• The data provide an insight on the growth potential of all male hybrids from interspecific hybridization of *O. karongae* male x *O. shiranus* females to improve fish yields in Malawi.
• The data form a basis for the formulation of a hypothesis on sex determination system of *O. shiranus* under the gene interaction model which is hitherto unknown.

## 1 Data Description

### 1.1 Data

There are two datasets, the first dataset comprises Table 1a, Table 2a, Table 2b to 4. These tables present data on fish body weights from four SETs of an experiment. Table 1 is for SET 1, that is, body weight data of progeny from interspecific mating of *O. karongae* (male) x *O. shiranus* (female). Table 2 is for fish body weights that were obtained from SET 2 of the experiment, which is the progeny of *O.shiranus* pure crosses that were spawned in pond based hapa 1. The fry were fed a sex reversal hormone (17α methyl-testosterone (MT) hormone) that was incorporated into a National Aquaculture Center formulated fry feed at the rate of 60mg/kg of the feed. The experiment duration was 28 days, starting from the yolk absorption stage. Table 3 presents data for fish in SET 3, which were also progeny from *O. shiranus* pure crosses but was spawned in an indoor re-circulatory hatchery at a controlled temperature of 27°C. This SET was a control group for spawning temperature of the pond based hapa fish in SET 2. The fish in SET 3 were also fed 17α MT hormone incorporated feed at 60kg/kg of feed for 28 days. Table 4 presents data from *O. shiranus* pure strain that was collected from a pond based breeding hapa 1 and was fed a normal National Aquaculture Center formulated fry feed that was not incorporated with any sex reversal hormone (Control group for the artificial sex reversed fish). There are six data tables within each SET, which represent the day that the body weight was recorded after stocking. Table numbers represent SET numbers and are accompanied by a letter script from a to f. Table numbers accompanied by a letter ‘a’ represent fish body weights in that SET that were recorded on the initial stocking day, which is the first day of the experiment. Body weight table numbers within a SET that were recorded fourteen days after stocking have a letter b, while those recorded twenty-eight days after stocking have a letter c. Body weights that were recorded on the 42^nd^ day of the experiment, their table numbers are accompanied by letter d. Body weights that were recorded on the 56^th^ and 70^th^ days of the experiment, have table numbers that are accompanied by letters e and f respectively, within each SET.

The second dataset is presented as Table 5. These are data on sex ratio determination from a sample of 80 fish that was obtained from three replicates within each SET (n=320). This fish was sacrificed on the 70^th^ day of the growth experiment and their gonads were removed and stained with aceto-carmine solution. Sex was determined with the aid of a microscope at x100 and x400 magnifications. SET 1 is for progeny from interspecific mating of *O. karongae* (male) x *O. shiranus* (female); SET 2 is progeny from *O.shiranus* pure crosses in hapas and fed 17α MT hormone incorporated feed; SET 3 is progeny from *O. shiranus* pure crosses in re-circulatory hatchery at 27°C (control group for spawning temperature and was also fed 17α MT hormone incorporated feed) and SET 4 is progeny from *O.shiranus* pure crosses in a hapa and was reared on normal feed without the 17α MT hormone (Control).

## 2 Experimental Design, Materials and Methods

### 2.1 Source of Broodstock

The experiment was conducted at National Aquaculture Centre (NAC), Domasi, Zomba district in Malawi, from February to May, 2019. Broodstock of mean body weight (MBW±1SE) of 110±0.3g for males and 105±0.54g for females of *O. karongae* and *O. shiranus* were selected from earthen fish breeding ponds at the NAC. The selected fish were healthy and with ripe gonads and was originally collected in 2017 from the South East Arm of Lake Malawi at Makawa beach in Mangochi district. The fish was stocked in separate breeding earthen ponds at the NAC.

### 2.2 Fry production

Two breeding hapas of 3 × 3 × 1 m^3^ were inserted in an earthen pond of 1,200m^2^. In the first hapa, ripe and healthy *O. karongae* male *and O. shiranus* female broodstock was stocked at a density of 2 fish /m^2^ and a sex ratio of 1:2 (male: female). In the second hapa, ripe and healthy *O. shiranus* males and females were stocked at the same density and sex ratio as in hapa 1. Thus, there were 18 males and 36 females in each hapa. According to Baroiller and D'Cotta [1] and Cnaani et al. [2] the success of hormonal sex reversal using 17α methyl testosterone (17α-MT) also depends upon spawning temperature. To control for the breeding temperature, males and females of *O. shiranus* were stocked in a 9 m^3^ circulatory tank in an indoor hatchery in which the water temperature was maintained at 27°C. The fish stocking density was 2 fish/m^2^ and the sex ratio was 1:2 (male: female). The females in the hapas and tank were checked if they were brooding eggs after 14 days and fertilized eggs were collected from the mouths of brooding females. The eggs from the females in the breeding hapas and indoor hatchery tank were incubated in brooding bottles with constant flow of water in the indoor hatchery at 27°C until they hatched. After yolk absorption, at swim up, fry from the two breeding hapas and the indoor hatchery tank were stocked in 20m^2^ re-circulatory tanks in the indoor hatchery at NAC for sex reversal experiments.

### 2.3 Sex reversal treatments and control groups

There were four batches of fry, each being a separate SET of an experiment and each SET was replicated three times (Table 1a, Table 2a, Table 3a to 4). Each replicate had 70 fish giving a total of 210 fish per treatment (Table 1a, Table 2a, Table 3a to 4). SET 1 comprised the hybrid fry that was pooled together from five females when *O. karongae* male was crossed with *O. shiranus* female in breeding hapa 1 (Table 1). SETs 2 and 4 comprised fry from ten females during pure crossing of *O. shiranus* from the breeding hapa 2 (Tables 2 and 4). Finally, SET 3 was pure cross of *O. shiranus* (pooled together from six females that bred in the re-circulatory indoor hatchery tank). The fry in all the SETs were fed a standard NAC fry feed comprising 38% crude protein at 3% body weight per day, given five times a day, every two hours from 08:00 to 16:00 in equal amounts. However, the feed for the fry in SETs 2 and 3 was incorporated with 17α- methyl testosterone (MT) hormone at the rate of 60 mg / kg of feed [3]. Therefore, SET 3 was a control group for spawning temperature of the hapa spawned and 17α-MT hormone sex reversed *O. shiranus* purebred group. SET 4 was a control group for the 17α-MT hormone sex reversal treatment (Table 3). After 28 days, the fry from each SET was transferred to rearing hapas of 3 × 3 × 1m^3^ that were installed in earthen ponds. Within each of the three replicates in each SET, the fry was fed a standard NAC floating fry feed for 70 days from the day that the fish were transferred to the rearing hapas (Table 4).

### 2.4 Growth testing of the hybrids and sex reversed pure bred fish

The body weight of the fish in the rearing hapas were recorded every 14 days for the whole 70-day rearing period (Tables 1 to 4). On the 70^th^ day, all the fish in each replicate and SET was weighed to the nearest 0.1g and the time series fish body weight data are presented in Tables 1 to 4.

### 2.5 Sex determination of fingerlings

On the 70^th^ day, a random sample of 80 fish of (MBW±1SE) of 8.05±0.46g was collected from each SET and was sacrificed to determine their sex. Two replicates within each SET contributed 27 fish, while the third replicates each contributed 26 fish (Table 5). The sex of the fish was determined using Aceto –carmine staining method after Guerrero and Shelton [4] and Menu et al. [5]. The Aceto-carmine solution was prepared and stored in the laboratory after Guerrero and Shelton [4]. The stained gonads were easily identified because they were in contact with the ventral portion of the swim bladder. The gonadal tissue was removed using a pair of fine forceps and then mounted on glass slide and crushed. Five drops of Aceto –carmine solution were added to the crushed gonads on the glass slide. The crushed gonads were then examined using a compound microscope at 100 x and 400x magnifications [4] to determine their sex (Refer to Figures 1 and 2 in the main journal article at: https://doi.org/10.1016/j.aqrep.2020/100274. Data on the sex ratios is presented in Table 5.

### 2.6 Data analysis

Data were analyzed using SPSS version 20.0. Sex ratio from the control group was tested from the expected 1:1 (male: female) ratio using Chi-square goodness of fit test at the 5% test level of significance. The differences in the percentages of males among the treatments were tested by the use of comparison of proportions method in SPSS, using Chi-square test in the Legacy Dialogs of the Nonparametric tests at the 5% test level of significance. Deviations of the observed sex ratios in the interspecific hybrids from the expected ratios according to the gene interaction model reported by Hammerman and Avtalio [6] were determined using Chi-square goodness of fit test at the 5% test level of significance. One-way Analysis of Variance (ANOVA) was used to compare differences among the mean stocking and mean final body weights of the fish from each SET at the 5% test level of significance. Significantly different means were separated using Duncan's multiple range test in the SPSS software at the 5% level of significance.

Table 1a, Table 1b, Table 1c, Table 1d, Table 1e, Table 1f, Table 2a, Table 2b, Table 2c, Table 2d, Table 2e, Table 2f, Table 3a, Table 3b, Table 3c, Table 3d, Table 3e, Table 3f, Table 4a, Table 4b, Table 4c, Table 4d, Table 4e, Table 4f, Table 5

**Table 1a:** Body weight data from SET 1 of the experiment, i.e., Progeny from interspecific mating of *O. karongae* (male) x *O. shiranus* (female) that was collected on the initial stocking day, i.e., day 1.

Fish No.	Replicate 1 BW (g)	Replicate 2 BW (g)	Replicate 3 BW (g)	Fish No.	Replicate 1 BW (g)	Replicate 2 BW (g)	Replicate 3 BW (g)
1	1.9	2	2.3	36	2.3	2	2.3
2	1.8	2	2.4	37	2.2	2.2	2.1
3	1.6	2.2	2	38	2.1	2.1	1.9
4	2.2	2.1	2.1	39	2.2	2	1.8
5	2.1	2	1.9	40	2.4	2	2
6	2	2.1	1.8	41	2.5	2	2
7	2.3	2.4	2.4	42	2.1	2.5	2.4
8	2.4	2.5	2.3	43	2	2.1	1.9
9	2.3	2.4	2.5	44	2	2.3	2.1
10	1.9	2	2.3	45	2.2	2.2	2.2
11	2.3	2	2.2	46	2.2	2	2.2
12	2.4	2.3	2.1	47	2.4	2	2.1
13	2.4	2.1	2.1	48	2.3	1.9	2
14	2.3	2.2	2.2	49	2.2	2.2	2
15	2.4	2.1	2.3	50	2.2	1.9	2
16	1.9	2	2.1	51	2.3	2.1	2.5
17	1.7	2.3	2.2	52	2.2	2.2	2.4
18	1.8	1.9	2	53	2.1	2.4	2.3
19	1.9	2	2	54	2.2	2.3	2.2
20	2	2.3	1.9	55	2.3	2.1	2.1
21	2.1	2.2	2.3	56	2.1	2.2	2.1
22	2.2	2.2	2.2	57	2.2	2.2	2.3
23	2.3	2.2	2.1	58	2.3	2.4	2.2
24	2.4	2.3	2.2	59	2.4	2.4	2.4
25	2.3	2.4	2.3	60	2.1	2.7	2.3
26	2.2	2.3	2.4	61	2.2	2.1	2.2
27	1.8	2	2.2	62	2.3	2	2
28	1.8	2	2.1	63	2.3	1.9	2.1
29	1.8	2.2	1.9	64	2.2	2.3	2.2
30	1.9	2	1.8	65	2.1	2	2
31	1.7	2	2.1	66	1.9	2.1	2.4
32	2.3	2	2.1	67	2	2	2.3
33	2.2	2.3	2.2	68	2.4	2.3	1.9
34	2.2	2.4	2.2	69	2.1	2.2	2
35	2.7	2	2.2	70	2.3	2	2.3

**Table 1b:** Body weight data from SET 1 of the experiment, i.e., Progeny from interspecific mating of *O. karongae* (male) x *O. shiranus* (female) that was collected on the 14^th^ day after stocking.

Fish No.	Replicate 1 BW (g)	Replicate 2 BW (g)	Replicate 3 BW (g)	Fish No.	Replicate 1 BW (g)	Replicate 2 BW (g)	Replicate 3 BW (g)
1	1	5	5.3	36	4.5	4.3	4.4
2	2	4.8	5	37	4.9	4.4	4.5
3	3	4.7	4.8	38	4.9	5.1	5
4	4	4.8	4.9	39	4.8	4.7	4.5
5	5	4.9	4.7	40	4	4.3	4.7
6	6	4.8	5	41	4	4.8	5
7	7	4.9	4.8	42	4.9	4.9	4.8
8	8	4.7	5.2	43	5	5.2	4.3
9	9	4.5	4.8	44	4.7	4.1	4.9
10	10	5	4.8	45	4.8	4.6	4.8
11	11	4.9	4.9	46	4.9	4.5	4.8
12	12	4.8	5.1	47	5	4.9	4.8
13	13	4.6	5.2	48	5	5	4.6
14	14	4.9	5	49	4.9	5.2	4.7
15	15	4.8	4.9	50	4.8	5.3	5
16	16	4.9	4.8	51	4.7	5.1	5
17	17	5	4.7	52	4.8	4.9	4.8
18	18	4.8	5	53	5.3	4.8	4.7
19	19	4.8	4.7	54	5.2	4.7	4.8
20	20	4.9	4.5	55	5.1	4.6	4.8
21	21	4.8	5.1	56	4.6	4.8	4.9
22	22	5	4.8	57	4.7	4.9	4.7
23	23	5	4.8	58	5.4	4.2	4.4
24	24	4.9	4.6	59	5.3	4.7	4.7
25	25	4.6	4.9	60	5.2	4.9	4.9
26	26	4.8	4.3	61	5.1	5.3	5.1
27	27	5.4	4.9	62	4.8	5.1	5
28	28	5.5	4.8	63	4.9	5.2	5
29	29	5.2	4.7	64	4.9	4.9	4.8
30	30	5.1	5	65	4.9	4.8	4.7
31	31	4.9	5.2	66	4.8	5.2	5.1
32	32	4.8	5.1	67	4.4	5.1	5
33	33	4.7	5.3	68	4	5.3	5.2
34	34	4.8	4.8	69	4.9	4.8	4.9
35	35	4.9	4.7	70	4.7	5.2	4.7

**Table 1c:** Body weight data from SET 1 of the experiment, i.e., Progeny from interspecific mating of *O. karongae* (male) x *O. shiranus* (female) that was collected on the 28^th^ day after stocking.

Fish No.	Replicate 1 BW (g)	Replicate 2 BW (g)	Replicate 3 BW (g)	Fish No.	Replicate 1 BW (g)	Replicate 2 BW (g)	Replicate 3 BW (g)
1	5.5	5.3	5.2	36	5.7	4.8	5.1
2	5.3	5.2	5.1	37	5	4.9	5.1
3	5.2	5.1	5	38	5.2	4.8	5
4	5.5	5	5.1	39	5.4	5.1	5.1
5	5.1	5	5.2	40	5.6	4.9	5.2
6	5.2	4.9	5.3	41	5.7	4.9	5.1
7	5.3	4.8	5.2	42	5.8	4.8	5
8	5.2	5	5.1	43	5.3	5	5.2
9	5.1	5	5.2	44	5.2	4.7	5.5
10	5.2	5	5.1	45	5.1	4.6	5
11	5.1	5.6	5.3	46	5.4	4.7	5.2
12	5.4	5.4	5	47	5.6	4.9	5.3
13	5.3	5.3	5	48	5.3	4.8	5.4
14	5.3	5.3	5.1	49	5.2	4.7	5
15	5.4	5.2	5	50	5.5	4.5	5.2
16	5.2	5.1	4.9	51	5.6	4.8	5.1
17	5.1	5.3	4.8	52	5.7	4.9	5.3
18	5.4	5.3	4.7	53	5.8	4.8	5.5
19	5.1	5.2	4.6	54	5.7	5	5.2
20	5.2	5.1	4.9	55	5.5	4.7	5.1
21	5.7	4.8	5.1	56	5.3	4.8	5.5
22	5.2	5.5	5.2	57	5.5	4.7	5.4
23	5.5	5.3	5	58	5.4	4.5	5.1
24	5.4	5.2	5.2	59	5.3	5	5.2
25	5.2	5.1	5.1	60	5.4	5	5.1
26	5.1	5	5	61	5.5	4.8	5.3
27	5.5	4.9	5	62	5.7	4.7	5.5
28	5.4	4.8	4.8	63	5.6	5	5.6
29	5.6	5.1	4.7	64	5.4	5	5.2
30	5.1	4.7	4.9	65	5.6	5	5.2
31	5.6	5.2	5	66	5.7	4.9	5.2
32	5.3	5.1	5.1	67	5.3	4.8	5
33	5.2	5	5.2	68	5.6	4.7	5
34	5.6	4.9	5.3	69	5.5	4.7	5.1
35	5.7	4.9	5.0	70	5.4	4.7	5.2

**Table 1d:** Body weight data from SET 1 of the experiment, i.e., Progeny from interspecific mating of *O. karongae* (male) x *O. shiranus* (female) that was collected on the 42^nd^ day after stocking.

Fish	Replicate 1 BW (g)	Replicate 2 BW (g)	Replicate 3 BW (g)	Fish	Replicate 1 BW (g)	Replicate 2 BW (g)	Replicate 3 BW (g)
1	9.2	9.8	10.1	36	8.4	10.3	9.7
2	9	10	9.8	37	9	10.1	9.2
3	8.7	10.1	9.9	38	9.3	9.9	9.3
4	9.2	10.3	9.1	39	9.2	9.8	9.8
5	9.1	10.4	9.5	40	9.1	10.2	9.9
6	9.3	9.9	9	41	9	10.1	10.2
7	9.6	10.1	10.1	42	9	10.4	10.4
8	8.9	10.5	9.1	43	9	10.2	10.7
9	9	10.8	10.2	44	8.9	10.2	10.8
10	9.1	11.8	9.4	45	8.8	10.7	9.2
11	9.2	10.4	9.8	46	9	9.9	9.6
12	9.5	9.9	9.8	47	9	10.2	9.9
13	9	9.8	10.8	48	9.4	10.3	9.3
14	9.1	10.5	9.8	49	9.6	11.2	9.7
15	9.3	10.6	9.2	50	9.3	10.6	9.1
16	9.2	11.2	9.1	51	9.3	10.9	9
17	9.6	8.9	9.6	52	9.4	11.3	10.3
18	9.1	9.7	9.7	53	9.1	9.5	10.2
19	9.2	9.9	9.9	54	9.2	9.9	10.7
20	9.7	10.2	9.5	55	9.4	9.3	10.5
21	9.8	10.3	9.2	56	9.2	10.2	10.6
22	9.5	10.6	9.7	57	9.1	10.2	9.7
23	9.3	10.4	9.2	58	9	10.7	9.9
24	9.2	10.3	9.1	59	9	10.2	9.1
25	9.1	10.1	9	60	8.8	11.1	10.3
26	8.9	10	9	61	9.1	11.5	9.8
27	8.8	9.2	9.3	62	9.2	10	9.7
28	9.1	9.9	10.3	63	8.9	10.1	10.5
29	9.2	11.3	9.9	64	8.9	10.5	10.4
30	9.5	10.8	9.9	65	9	10.1	9.5
31	9.1	10.6	9.6	66	9	10.6	9.2
32	9.4	9.8	9.3	67	9	9.3	9.7
33	9.1	11.3	9.1	68	9.6	9.7	10.5
34	9.3	11.2	9.6	69	9.8	10.3	9.6
35	8.7	10.8	9.8	70	9.1	10.4	9.9

**Table 1e:** Body weight data from SET 1 of the experiment, i.e., Progeny from interspecific mating of *O. karongae* (male) x *O. shiranus* (female) that was collected on the 56th day after stocking.

Fish No.	Replicate 1 BW (g)	Replicate 2 BW (g)	Replicate 3 BW (g)	Fish No.	Replicate 1 BW (g)	Replicate 2 BW (g)	Replicate 3 BW (g)
1	10	10.8	10.7	36	10.3	11	10.1
2	9.8	11.5	10.6	37	10.2	11	10.5
3	9.9	11.3	10.5	38	10.3	11.7	10.2
4	9.8	10.9	11	39	9.9	10.9	10.2
5	9	10.7	11.3	40	9.8	10.8	10.1
6	9.2	11.2	11.6	41	9.7	10.7	10.5
7	9	10.8	10.2	42	10.4	11.8	9.9
8	9.5	11.5	10.9	43	10.2	11.9	9.7
9	9.9	11.1	10.8	44	10	10.7	9.1
10	9.1	10.9	11.2	45	9.7	11.4	10.4
11	9.8	10.8	10.4	46	9.9	11.2	10
12	9.9	11.2	10.3	47	10	10.9	10.8
13	10.1	11.8	10.9	48	9.2	11.2	10.8
14	10.8	11.5	10.7	49	9.5	11.3	10.6
15	9.8	10.9	10.9	50	9.5	11.1	10.5
16	9.7	10.5	11.7	51	9.8	11	10.3
17	9	11.1	10.3	52	10.1	11.8	10.5
18	10.8	11.7	11.3	53	9.9	11.2	10.2
19	9.9	10.8	10.9	54	9.3	10.8	10.6
20	9.8	10.6	11.2	55	9.2	11.5	10.2
21	10.9	11.1	10.3	56	9.9	11.4	9.9
22	10.8	10.9	10.7	57	9.8	11.2	9.8
23	9.4	11.3	11.9	58	10	10.7	9.7
24	9.8	11.5	10.8	59	10	11.1	10.1
25	9.9	10.9	10.3	60	10.1	11.2	10.3
26	10.1	10.9	10.5	61	9.6	11.5	10.4
27	10.2	10.7	10	62	9.9	11.5	10.6
28	10.6	11.9	10.6	63	10.2	11.3	10.3
29	10.3	11.2	11.9	64	10.3	11.9	10.6
30	9.8	11.7	11.7	65	10.4	11.2	10.7
31	9.9	10.9	10.1	66	10	11.1	10.9
32	9.1	10.8	10.4	67	9.9	11	10.6
33	9.2	11.5	10.2	68	10.2	11.6	10.8
34	9.8	11.2	10	69	10.6	11.2	10.4
35	9.7	11.1	10	70	9.6	10.9	10.3

**Table 1f:** Body weight data from SET 1 of the experiment, i.e., Progeny from interspecific mating of *O. karongae* (male) x *O. shiranus* (female) that was collected on the 70th day after stocking.

Fish	Replicate 1 BW (g)	Replicate 2 BW (g)	Replicate 3 BW (g)	Fish	Replicate 1 BW (g)	Replicate 2 BW (g)	Replicate 3 BW (g)
1	12	11.4	11.8	36	13.1	10.9	11
2	12.4	11.5	11	37	12.9	11.8	11.6
3	12	11.7	11.2	38	12.1	11	11.2
4	11.4	11.5	11.3	39	10.3	11.5	11.8
5	11.8	13	11.8	40	12.8	12	11.1
6	11.9	11.4	10.9	41	11.9	11	10.7
7	11.6	11.9	10.6	42	10.8	12.3	10.3
8	10.2	10.1	11.2	43	10.9	10.2	10.2
9	11.3	11.4	12	44	11.2	11	10.1
10	10.7	11	11.6	45	11.5	11	10.3
11	10.9	11.7	10.9	46	11.6	10.9	10.2
12	10.4	11.8	10.8	47	11.3	12	10.9
13	10.9	10.8	11.9	48	10.9	11.9	11.2
14	10.8	11.5	11.9	49	10.8	11.9	11.1
15	11.4	11.8	11.1	50	12.3	12	11
16	11.8	12	11.9	51	11.5	11.8	11.8
17	12.6	11.9	10.8	52	11.9	10.7	10.3
18	13.1	11.7	11.9	53	10.4	10.5	10.9
19	12.9	11.9	10.6	54	10.9	11.8	11.9
20	11	11.9	9.9	55	11.2	10.7	11.3
21	11.5	11	11.2	56	11.6	11	11.2
22	11.7	12	11	57	12	10.6	11
23	11.6	11.8	11.8	58	11.9	11.5	10.9
24	11	11.8	11.2	59	10.7	11.8	10.8
25	10.6	12	11.1	60	10.8	12.5	11.8
26	10.9	11	11	61	11.9	12	11.9
27	10.2	11.9	10.9	62	10.4	10.9	11.3
28	10.6	10.8	11.9	63	12.9	11.4	11.4
29	10.6	11.7	12.8	64	10.7	11.8	11
30	11.2	11.5	11.8	65	11.3	11.1	11
31	11.6	11.7	11.1	66	11.7	11.8	10.8
32	11.9	12	10.8	67	11.9	12	10.3
33	10.6	10.1	11.6	68	10.4	12	10.8
34	11.2	12	11.2	69	10.9	11.2	10.7
35	11.9	12	11	70	10.1	11	10.9

**Table 2a:** Body weight data from SET 2 of the experiment, i.e., Progeny from *O.shiranus* pure crosses in hapas and fed 17α MT hormone incorporated feed that was collected on the initial stocking day, i.e., day 1 of the experiment.

2: Time Series Data from SET 2							
Fish No.	Replicate 1 BW (g)	Replicate 2 BW (g)	Replicate 3 BW (g)	Fish No.	Replicate 1 BW (g)	Replicate 2 BW (g)	Replicate 3 BW (g)
1	2.2	2.1	2	36	2.1	1.9	2.1
2	2	1.9	2.3	37	2.2	1.8	2.2
3	2	2	2.2	38	2.1	2	2
4	1.9	2.1	2	39	2.1	2	2
5	1.8	2.1	2	40	2.1	2	1.9
6	1.9	2.2	2	41	1.9	2	1.8
7	2	2	2	42	1.8	2	2.1
8	2.2	2	2	43	1.7	1.9	2
9	2	2	1.8	44	2.2	2	2
10	2	2	1.9	45	2	2	2
11	2.3	2	1.8	46	2.1	2	2
12	2.2	2.3	2	47	2	2	2.1
13	2	2.4	2.1	48	1.9	2.2	1.9
14	2.1	2.1	2.2	49	1.8	2.3	1.7
15	1.9	2.1	2.3	50	2.2	2.1	1.9
16	2.1	2	2.2	51	2.1	2.1	2.2
17	2.2	2	2	52	2	1.8	2
18	2.3	2	2	53	2	1.9	2
19	1.8	2	2	54	2	2.1	2.1
20	1.7	2	2.1	55	2	2.3	2
21	1.9	1.9	2.3	56	2	2.2	2.1
22	2.2	1.8	1.9	57	2	2.2	2.3
23	2	1.7	2	58	2	2.1	2.1
24	2	2	2.2	59	2	1.9	1.9
25	2	2	1.9	60	2	1.8	1.9
26	1.9	2	1.8	61	1.9	2	2.1
27	1.8	2	1.7	62	1.9	2	2.1
28	1.7	2	2.2	63	1.7	2	2.1
29	2.2	2.1	2	64	1.8	2	1.9
30	2	2.2	2.1	65	2.1	2.2	1.8
31	2	2	2.3	66	2.3	2.2	2
32	2.2	2.1	2.2	67	2.4	2.3	2.2
33	2.1	2	1.9	68	2.1	1.9	2.3
34	2	2.1	1.8	69	2	1.9	2.2
35	2	1.9	1.7	70	1.9	1.8	2.1

**Table 2b:** Body weight data from SET 2 of the experiment, i.e., Progeny from *O. shiranus* pure crosses in hapas and was fed 17α MT hormone incorporated feed that was collected on the 14th day after stocking.

Fish No.	Replicate 1 BW (g)	Replicate 2 BW (g)	Replicate 3 BW (g)	Fish No.	Replicate 1 BW (g)	Replicate 2 BW (g)	Replicate 3 BW (g)
1	3	3.4	3.5	36	3.2	2.8	3.2
2	3.2	3.2	3.2	37	2.7	2.9	3.4
3	3.1	3.3	3.1	38	3	3.2	3.1
4	2.9	3	3.3	39	3.3	3.1	3.3
5	3.4	3	3.5	40	3	3.3	3.6
6	3	2.9	3.2	41	3.1	3.4	3.9
7	3.4	3.1	3.1	42	3.2	3.6	2.8
8	2.9	3.2	3	43	3	3.5	2.9
9	3.3	3.6	3	44	3.3	3.1	3.1
10	2.9	3.5	3.5	45	3.2	3.2	3.2
11	3.2	3.4	3.7	46	3.1	2.8	3.4
12	3.1	3.2	3.8	47	3.2	2.9	3
13	3.3	4	3.2	48	3	3.2	3.1
14	3	4.1	3.8	49	3.6	3.3	3.6
15	3.4	3.8	3.2	50	3	3.1	3.5
16	3.2	3.6	3.3	51	3	3.2	3.3
17	3.1	3.3	3.1	52	3.1	3.5	3.9
18	3.2	3.3	2.6	53	3.2	3.6	3.2
19	3.3	3.4	2.7	54	3.3	3.1	3.7
20	3.4	3.2	2.8	55	3.1	3.2	3.8
21	2.9	3.3	3.9	56	3	3.5	3
22	3.2	3	3.8	57	3.6	3.3	3.1
23	3.3	3	3.2	58	3	3	2.9
24	3	3	3.4	59	3	3	2.8
25	3	3.1	3.6	60	3.1	3	3.2
26	2.9	3.2	4.1	61	3.2	3.2	3.6
27	3.1	3.5	4.2	62	3.3	3.3	3
28	3.2	3.3	3.3	63	3.2	3.4	3.5
29	3.3	3.2	2.7	64	3.3	3.5	3.3
30	3.1	3.1	2.6	65	3.1	3.2	3.2
31	3	3.3	2.5	66	3.2	3.6	3.1
32	3	3.1	3.2	67	3.6	4	2.9
33	3	3.2	2.9	68	3.1	4.2	2.7
34	3.3	3.2	2.8	69	3.1	4.1	3.6
35	3.1	2.9	2.7	70	3.6	4.4	3.1

**Table 2c:** Body weight data from SET 2 of the experiment, i.e., Progeny from *O. shiranus* pure crosses in hapas, fed 17α MT hormone incorporated feed that was collected on the 28th day after stocking.

Fish No.	Replicate 1 BW (g)	Replicate 2 BW (g)	Replicate 3 BW (g)	Fish No.	Replicate 1 BW (g)	Replicate 2 BW (g)	Replicate 3 BW (g)
1	4.4	5.9	5	36	4	4.7	5
2	4.5	5.5	4.6	37	4.2	4.7	5.2
3	5	5.7	4.7	38	4.1	4.6	5.7
4	5.7	5.2	4.3	39	3.9	5.1	5
5	3.2	5.8	4.2	40	3.8	5.7	4.5
6	4.2	4.8	5	41	3.7	5	4.7
7	3.6	4.9	5.4	42	3.6	5.3	4.9
8	3.9	5	5.2	43	4.3	5.1	5
9	4.1	4.9	5.1	44	4.2	5	5.1
10	4.5	4.8	5	45	4.1	4.9	4.8
11	4.1	5.2	5	46	4	5	5
12	4.6	5.1	4.5	47	3.9	5.4	5.1
13	4.4	5.2	4.8	48	3.8	5.8	4.7
14	4.2	5.7	4.9	49	3.7	6	4.3
15	4.3	5.9	5.2	50	3.4	5.3	4.6
16	4.1	5.1	5.2	51	4	5.4	4.2
17	4.2	5.4	4.9	52	4.4	5.8	4.1
18	5	4.2	4.8	53	4.3	5.3	4.4
19	5.2	4.2	4.5	54	4.1	5.2	4.8
20	3.6	4.1	5.2	55	4.2	5	4.7
21	3.9	5	5.2	56	4.1	5	4.1
22	5.2	4.9	5.3	57	3.9	5.2	3.9
23	4.9	4.8	4.6	58	3.6	5.5	3.8
24	4.7	4.8	4.1	59	4.5	5	4
25	4.8	4.7	4	60	4.1	5	4.7
26	5.1	4.6	4.8	61	4.2	5.2	5
27	5.3	5.6	4.9	62	4.1	5.5	5.3
28	5.2	5.9	5.1	63	4	5.6	5
29	5.6	5.5	4.9	64	4	5.3	4.9
30	5.2	5.4	4.8	65	4.1	5.2	4
31	4.8	5.4	4.3	66	4	5.1	5
32	4.9	5.2	4.8	67	4	5	4.6
33	4.1	5.1	4.2	68	4.7	5.7	4.8
34	4.2	4.9	4.1	69	4.9	4.8	4.7
35	3.9	4.8	4.7	70	4.9	4.1	4

**Table 2d:** Body weight data from SET 2 of the experiment, i.e., Progeny from *O. shiranus* pure crosses in hapas and was fed 17α MT hormone incorporated feed that was collected on the 42th day after stocking.

Fish No.	Replicate 1 BW (g)	Replicate 2 BW (g)	Replicate 3 BW (g)	Fish No.	Replicate 1 BW (g)	Replicate 2 BW (g)	Replicate 3 BW (g)
1	7.8	6.4	7.1	36	8	6.2	6.9
2	8.1	6.3	7.2	37	8.2	6.1	7.1
3	8.2	6.2	7.4	38	8.1	6.7	8.2
4	8.3	7.2	6.9	39	8.2	6.4	7.4
5	8.1	7.3	6.8	40	8.1	6.3	7.2
6	8.2	7.7	7.1	41	8.2	6.2	7.1
7	7.8	6.2	7.3	42	7.5	6.1	7.3
8	7.9	8	7.2	43	7.7	6.2	6.9
9	8.1	8.2	7.4	44	7.9	6.7	9
10	8.2	8.1	7.8	45	8	6.8	7.9
11	7.4	7.5	6.9	46	8.2	6	7.1
12	7.6	6.2	6.6	47	8.3	6	6.2
13	7.7	6.1	6.5	48	8.1	6.1	7.3
14	7.9	6.2	6.9	49	8	6	6.3
15	7.2	6.3	6.1	50	8.3	5.9	6.5
16	8	6.8	6.5	51	7.9	5.8	6.1
17	8.2	6.1	7.3	52	8.6	5.8	7
18	8.4	6.3	7.7	53	7.8	5.7	7.1
19	8.5	6.1	6.8	54	7.7	6.3	6.8
20	8.6	5.8	5.3	55	6.9	6.7	8
21	7.7	7.1	5.2	56	7	6.1	9
22	7.5	7.2	5.6	57	7.5	6.2	8.9
23	8.1	6.9	6.1	58	7.9	6.3	7
24	8	6	6.9	59	8.2	6.8	8.4
25	7.7	6	6.9	60	7.9	6.6	9
26	7.5	6.3	7	61	8.2	6.6	7.2
27	7.2	6.8	6.2	62	9.1	6.7	8
28	7	6.2	6.3	63	7.9	6.3	8.3
29	7.1	6.3	5.9	64	5.8	6.2	8
30	7.3	6	6.1	65	6.8	6.1	8.9
31	7.5	7	6	66	7.7	6.3	8.2
32	7.4	6.9	6.9	67	6.9	6.2	8.6
33	7.2	6.6	6.2	68	7.2	5.7	8
34	7.8	6.5	6.7	69	7.1	5.4	7.8
35	7.9	6.3	6	70	8	5.3	7

**Table 2e:** Body weight data from SET 2 of the experiment, i.e., Progeny from *O. shiranus* pure crosses in hapas and was fed 17α MT hormone incorporated feed that was collected on the 56th day after stocking.

Fish No.	Replicate 1 BW (g)	Replicate 2 BW (g)	Replicate 3 BW (g)	Fish No.	Replicate 1 BW (g)	Replicate 2 BW (g)	Replicate 3 BW (g)
1	8	8.2	8.5	36	8.4	7.9	8
2	8	8.1	7.7	37	8.2	7.6	7.9
3	8	7.8	7.6	38	8	8.1	7.1
4	7.8	8.1	7.9	39	7.2	8.3	8.2
5	7.5	8.3	8	40	8.2	8.4	7.6
6	8	7.6	8.1	41	8.6	8.5	8.6
7	8.7	7.1	7.5	42	7	8.2	8.2
8	8.3	7.2	8.2	43	8	7.9	8.1
9	8.1	8	8.1	44	8.1	7.6	8.7
10	8	8.1	8	45	8.5	7.9	8.1
11	7.9	7.8	7.7	46	8.8	7.8	7.9
12	8.3	7.6	7.3	47	8	8.3	7.8
13	8	8	7.9	48	7.8	8.4	8.7
14	8.3	8.2	8.4	49	8.2	8.8	8.3
15	8	8.3	8.5	50	8	7.8	8.2
16	8.2	7.6	7.8	51	8	7.9	7.6
17	8	7.8	7.9	52	7.9	8.2	7.9
18	7.1	8.1	8.2	53	8	8.1	7.6
19	8.1	8.3	8.4	54	8.4	8	7.5
20	7.9	8	8.3	55	7	8.3	7.3
21	8.3	7.9	8.1	56	8.2	8.5	8
22	8.7	7.8	7.7	57	8.5	7.1	8.3
23	8.6	7.7	7.9	58	8.3	7.3	8.2
24	7	8.2	7.6	59	8.5	7.4	8.1
25	8.4	8.3	8.1	60	8	7.6	8.3
26	9	8.1	8.2	61	8	7.3	7.8
27	7	8	8.3	62	8.3	7.2	7.9
28	8	7.9	7.9	63	7.1	7	7.1
29	8.1	8.1	8	64	8.1	7.6	8.2
30	7.3	7.6	7.2	65	8	7.3	7.6
31	8	7.8	7.7	66	8.5	7.7	7.7
32	8	8.1	8.1	67	8.7	7.1	7.9
33	8.8	8.2	8.5	68	8	7.2	8.3
34	8.7	8.2	7.2	69	7.2	7.1	8.5
35	7	7.9	7.5	70	7	8.1	8.1

**Table 2f:** Body weight data from SET 2 of the experiment, i.e., Progeny from *O. shiranus* pure crosses in hapas and was fed 17α MT hormone incorporated feed that was collected on the 70th day after stocking.

Fish No.	Replicate 1 BW (g)	Replicate 2 BW (g)	Replicate 3 BW (g)	Fish No.	Replicate 1 BW (g)	Replicate 2 BW (g)	Replicate 3 BW (g)
1	9	8.6	9.2	36	9	8.3	8.2
2	8.1	8.7	8.9	37	8.7	8.2	8.9
3	8.5	9	8.2	38	9	8.5	9.5
4	8.7	8.9	9.4	39	8.2	9	9.4
5	8.3	9.2	8.9	40	8.9	8.9	8.7
6	8.1	8	9.1	41	8.7	8.6	8.8
7	8	8.3	9	42	9	8.8	8.5
8	8	8.8	9.3	43	8.1	8.1	8.5
9	7.9	8.7	8.9	44	8.7	9	9
10	7.8	8.9	8.8	45	8.6	8.5	8.9
11	7.7	8.3	8.7	46	8.9	9.3	9.2
12	7.5	8.6	8.8	47	8.9	8.9	9.5
13	8	8.3	8.3	48	8	9	9.4
14	8.9	8.9	8.9	49	8.9	8.7	9
15	8.2	9.1	8.7	50	8.8	8.9	9.2
16	8.3	9	9	51	8.2	9	8.2
17	8.7	8.2	8.9	52	8.1	8.9	9
18	8.8	8.9	8.7	53	8	8	8.7
19	8.3	8.8	8.2	54	8.5	8.9	8.9
20	8.1	8.1	8.8	55	8.7	9	9.2
21	8	8.7	9.1	56	8.9	8.2	9.1
22	8.7	8.8	9.3	57	8.6	8.1	8.3
23	8.8	8.7	8.8	58	8.2	9.5	9.4
24	8.4	8.3	9	59	8.1	9.6	8.7
25	8.9	8.6	8.9	60	8	9	8.2
26	8.2	9	8.2	61	8.7	9.2	9.1
27	8.7	8.9	8.9	62	8.2	8.9	10.5
28	8.8	8.3	9.2	63	8.6	9	9.1
29	8.9	8	9.8	64	8.9	8.9	8.9
30	8.8	8.2	9.1	65	8.3	9.1	9.1
31	8.9	9.1	8.8	66	8.8	8	8.7
32	8.3	8.2	8.7	67	8.1	8.4	8.5
33	9	8.1	8.3	68	9	8.3	8.9
34	8.5	8.5	9.2	69	9.1	9.5	8
35	8.8	8.4	8.8	70	9	9.4	8.7

**Table 3a:** Progeny from *O. shiranus* pure crosses that was spawned in re-circulatory hatchery at 27°C (control group for spawning temperature but fed 17α MT hormone incorporated feed) (SET 3). The data was collected on 1^st^ day of the experiment

3: Time Series Data from SET 3							
Fish No.	Replicate 1 BW (g)	Replicate 2 BW (g)	Replicate 3 BW (g)	Fish No.	Replicate 1 BW (g)	Replicate 2 BW (g)	Replicate 3 BW (g)
1	2.1	1.9	2.2	36	2.2	2.3	2.1
2	2.2	2.3	2.2	37	2	2.1	2.4
3	2.3	2.2	2.3	38	2.2	2.4	2.6
4	2.5	2.1	2.4	39	2.3	2.5	2.7
5	2	2	1.9	40	2.1	2.2	2.3
6	1.9	2	1.9	41	2	2.1	2.1
7	1.8	2	1.8	42	2	2.4	2
8	1.9	2	2.3	43	2.3	2.3	2.3
9	1.9	2.1	2.1	44	2.1	2.2	1.8
10	2	2	2.2	45	2.6	2.1	1.8
11	2.2	2.1	2.4	46	2.1	2.1	2.1
12	2	2	2.1	47	2.4	2	2.1
13	2	2.1	2	48	2.1	2	2
14	2	2.3	2.1	49	2.2	2.2	2
15	2.1	2.5	2	50	2.1	2.1	1.9
16	2.2	2.3	2.3	51	2.4	2	2
17	2.3	2.2	2.4	52	2.3	2	1.9
18	2	2.1	1.9	53	2	2.1	2
19	2.1	2	2	54	2.1	2.1	2.3
20	2.1	2	2.1	55	2.3	2.2	2.1
21	2	2.1	2.2	56	2.2	2.1	2.2
22	1.9	2.4	2.4	57	2.1	1.9	2
23	2.2	2.2	2.3	58	2.1	2	2.1
24	2.1	2.2	2.1	59	2	2.1	2.2
25	2	2.4	2	60	1.8	2.2	2.1
26	2.4	2.1	2	61	2.2	2.3	2.3
27	2.3	2	1.9	62	2.4	2	2.4
28	2	2.1	2.3	63	2.3	2	2
29	2	2.1	1.8	64	2.3	2.1	2.1
30	2.1	2.2	2.2	65	2.1	2.2	2
31	2.5	2.4	2.3	66	2	2.1	1.9
32	2.1	2	2.2	67	2.2	2.2	2.1
33	2	2.1	2.2	68	2.1	2.2	2.2
34	2.1	2.2	2.1	69	2.2	2.1	2.3
35	2.4	2.5	2.3	70	2.3	2	2

**Table 3b:** Progeny from *O. shiranus* pure crosses that was spawned in re-circulatory hatchery at 27°C (control group for spawning temperature but fed 17α MT hormone incorporated feed). The data was collected on 14^th^ day of the experiment (SET 3)

Fish No.	Replicate 1 BW (g)	Replicate 2 BW (g)	Replicate 3 BW (g)	Fish No.	Replicate 1 BW (g)	Replicate 2 BW (g)	Replicate 3 BW (g)
1	3.2	3.6	4	36	3.1	3.2	3.6
2	3.4	3.5	3.5	37	2.9	4	3.9
3	3.5	4.1	3.8	38	2.8	3.5	3.8
4	3.2	3.6	3.9	39	3.1	3.6	3.9
5	3	3.8	4.1	40	3.6	3.3	4
6	3.4	3.7	3.7	41	3.1	3.6	3.4
7	3.6	3.5	3	42	3.2	3.1	3.9
8	3.2	3.2	3	43	3.4	4	3
9	3.1	4.2	3.4	44	3.5	3.4	3.9
10	3	3.5	3.6	45	3.1	3.2	4
11	3.2	4.3	3.8	46	3.3	3.7	3.5
12	3	3.5	3.4	47	3.4	4.2	3.9
13	2.9	4.8	2.9	48	3.2	4	3.9
14	3.1	3.7	3.5	49	3.3	3.9	3.9
15	3	4	3.6	50	3.1	3.4	4
16	3.4	3.6	3.7	51	3.2	4.3	3.9
17	3.1	3.8	4	52	3.1	4.4	3.8
18	3.2	3.2	3	53	3.4	4.6	3.7
19	3	3.6	3.6	54	3.2	3.5	4
20	3.4	3.7	3.6	55	3.5	3.2	3.9
21	3.1	3.2	3.6	56	3.3	3.6	3.8
22	3.4	3.9	3.3	57	3	3.5	3.6
23	3.3	3.2	3.1	58	3.2	3.6	3.9
24	3.4	3.3	3.2	59	3.4	3.7	3.8
25	3.5	3.5	3.6	60	3.6	3.3	3.9
26	3.1	3.4	3.8	61	3.1	3.4	3.8
27	3	3.2	3.9	62	3.5	4.1	3.6
28	3.2	3.1	3	63	3	3.4	3.8
29	3.1	3.2	3.3	64	2.9	3.5	4.6
30	3	3.5	3	65	2.8	3.8	3.3
31	3.2	3.4	3.9	66	3.2	3.1	3
32	3.4	3.3	3.6	67	3.5	3.2	3
33	3.5	3.5	3.3	68	3.4	4	3.1
34	3.2	3.6	4.3	69	3.1	3.9	4
35	3	3.8	3.2	70	3	4.1	3.9

**Table 3c:** Progeny from *O. shiranus* pure crosses that was spawned in re-circulatory hatchery at 27°C (control group for spawning temperature but fed 17α MT hormone incorporated feed) (SET 3). The data was collected on 28^th^ day of the experiment

Fish No.	Replicate 1 BW (g)	Replicate 2 BW (g)	Replicate 3 BW (g)	Fish No.	Replicate 1 BW (g)	Replicate 2 BW (g)	Replicate 3 BW (g)
1	4.6	5	4.8	36	5	4.3	4.5
2	4.8	5.3	4.9	37	5.3	4.1	5.6
3	4.9	5.2	5	38	5.2	4.8	4.7
4	5	5.1	5.1	39	5.1	4.9	4.8
5	5.2	4.5	5.3	40	4.3	4.2	4.3
6	4.5	4.7	5.4	41	5.9	4.3	4.2
7	4.6	4.9	5.7	42	5	5.2	4.1
8	4.8	4.8	5.8	43	4.5	4.8	4
9	4.6	5.2	4.7	44	4.3	4.8	4
10	4.9	5.1	4.9	45	5.6	5.7	4.5
11	4.4	5.6	4.8	46	4.2	5.1	3.8
12	4.3	5.3	4.8	47	4.3	5.5	5
13	4.2	5.5	4.7	48	5.1	5.9	5.3
14	4.9	5.7	4.1	49	5.2	5.2	4.6
15	4.1	5.9	5.2	50	4.7	6.1	4.8
16	4.3	5.5	5.3	51	5.3	5.9	4.9
17	4.6	5.2	5.7	52	5	5.8	5
18	4.9	5.1	5.8	53	4.6	5.7	5.2
19	4.2	5.6	5.9	54	4.7	5.4	5.3
20	4.1	4.9	5.4	55	4.1	4.5	5.1
21	4	4.8	5.3	56	4.4	4.9	5.3
22	4.5	4.9	4.6	57	4.5	4.2	4.8
23	4.7	5.3	4.5	58	4	4.3	4.9
24	4.8	4.7	4.4	59	4.7	4.8	4.3
25	4.3	4.9	4.3	60	4.9	4.1	4.3
26	4.6	4.2	4.2	61	4.1	4	4.2
27	4.9	4.1	4.1	62	4.3	5	5.4
28	5	4.6	4.7	63	4.6	5.2	4.8
29	5.1	4.9	4.9	64	4.3	4.8	4.5
30	4	5.2	4.2	65	4.1	5.3	4.6
31	4.5	4.1	4.1	66	4	5.3	4.2
32	4.3	4.3	5	67	4.5	4.8	4.1
33	4.4	4.6	5.3	68	4.7	4.8	4.3
34	4.2	4	5.2	69	4.9	5.1	4.6
35	4.1	4.5	5.1	70	5	4.7	4.8

**Table 3d:** Progeny from *O. shiranus* pure crosses that was spawned in re-circulatory hatchery at 27°C (Control group for spawning temperature but fed 17α MT hormone incorporated feed) (SET 3). The data was collected on 42^th^ day of the experiment.

Fish No.	Replicate 1 BW (g)	Replicate 2 BW (g)	Replicate 3 BW (g)	Fish No.	Replicate 1 BW (g)	Replicate 2 BW (g)	Replicate 3 BW (g)
1	7.9	7.8	8	36	8.3	8.2	7.1
2	8.1	7.9	8.4	37	7.8	8.1	8.5
3	8.6	7.4	8.2	38	8.2	8.3	8.3
4	8.2	8.3	8.5	39	8.1	7.8	8.2
5	8.1	8.4	8.1	40	8.3	7.9	8.1
6	7.8	8.2	7.7	41	7.9	8.1	8.6
7	7.8	8.1	7.8	42	8.3	8.2	7.9
8	7.9	7.8	7.3	43	7.8	8.3	8
9	7.8	7.9	7.5	44	8.1	7.8	7.4
10	7.5	7.6	7	45	7.9	7.7	8.6
11	8.3	7.3	7.3	46	7.8	7.1	8.1
12	8.2	7.2	8	47	7.9	7.1	8.2
13	8.7	7.8	7.4	48	8.4	8.2	8.4
14	7.9	7	7.2	49	8.7	8.1	7.8
15	7.8	6.8	7.1	50	8.3	7.9	7.9
16	8.7	6.9	7.5	51	8.6	8	8
17	8.3	7.8	8	52	7.9	8.2	8
18	8.2	8	7.6	53	8.2	8.3	8.2
19	7.8	8.3	7.9	54	8	8.1	8.2
20	8.5	7.5	8.4	55	8	8.2	8
21	8.2	7.7	8.3	56	8.7	7.9	7.5
22	8.4	7.9	8.6	57	8.6	7	8.3
23	7.9	8	8.4	58	7.9	8.1	8
24	8.5	8.3	8.2	59	8.4	7.8	8.3
25	8.3	8.4	8.3	60	8.2	7.9	8.5
26	8.2	7	8.1	61	8.1	8.1	7.9
27	7.8	7.1	8.3	62	8.8	8	8.9
28	8	8	7.6	63	8.6	7	7.9
29	8.3	8.7	7.8	64	8.2	7	8.1
30	8	7.8	7.9	65	8.8	7.8	8.2
31	8.2	7.5	7.3	66	8.4	7.7	8.5
32	8.3	7.2	7.8	67	8.3	7.6	8.4
33	7.8	7.3	8.9	68	8.2	7.5	8.3
34	7.9	7.9	7.4	69	8	7.4	8
35	8.6	7.8	7.2	70	7.8	7.9	7.4

**Table 3e:** Progeny from *O. shiranus* pure crosses that was spawned in re-circulatory hatchery at 27°C (Control group for spawning temperature but fed 17α MT hormone incorporated feed) (SET 3). The data was collected on 56^th^ day of the experiment.

Fish No.	Replicate 1 BW (g)	Replicate 2 BW (g)	Replicate 3 BW (g)	Fish No.	Replicate 1 BW (g)	Replicate 2 BW (g)	Replicate 3 BW (g)
1	8.4	9	8.8	36	8.2	10.9	8.5
2	8.5	9.1	8.9	37	8.1	10.6	9.8
3	8.9	9.7	9	38	8.5	10.8	9.2
4	8.1	9.9	9.2	39	8.2	10.8	9.3
5	7.9	10	9.3	40	8.4	8.8	10
6	8.1	10.2	8.3	41	8.6	8.9	8.2
7	8.3	9.2	8.5	42	8.2	9.3	8.6
8	8.7	9.5	9.3	43	8.6	9.6	8.7
9	8.8	9.7	9.7	44	8.7	9.7	8.9
10	8.9	9	9.6	45	8.8	9.3	9
11	7.3	8.9	8.1	46	8.9	9	9.2
12	7.6	8.7	10.2	47	8.1	9.3	9.6
13	7.7	8.8	9.3	48	8.9	9.6	9.8
14	8.9	8.1	8.5	49	8.2	9.4	9.9
15	7.8	9	8.6	50	8.7	9.9	8.1
16	8.1	8.9	8.1	51	8.3	9.2	8.2
17	8	9.3	8	52	8.1	9	8.4
18	8.2	9.1	9.3	53	8.3	8.9	8.3
19	8.3	9.2	9.7	54	8.6	9.2	9
20	8.1	9.7	8	55	8.7	9.3	8.1
21	8.3	9.8	8.3	56	8.9	8.9	8.7
22	8.6	10	8.6	57	8.2	9.2	8.3
23	8.7	10.5	8.8	58	8.4	9.4	8.8
24	8.1	9.3	8.9	59	7.9	9.6	8.7
25	8.1	9.5	8.7	60	8.2	9.8	8.6
26	8.4	8.9	8.5	61	8.1	9.2	8.5
27	8.9	8.8	8.2	62	8.8	9.2	8.4
28	8	9	10.1	63	8	8.7	8.9
29	8.9	9.2	8.8	64	7.6	8.8	9.1
30	8.1	9.4	8.7	65	7.8	8.7	9
31	8.6	9.7	9	66	8	8.5	9.3
32	8.3	9.5	9.1	67	8.3	9.2	9.4
33	8.6	9.1	9.2	68	8.3	8.9	8
34	8.9	9.3	8.3	69	8.1	9	8.7
35	8.3	10	8.4	70	8	8.7	8.5

**Table 3f:** Progeny from *O. shiranus* pure crosses that was spawned in re-circulatory hatchery at 27°C (control group for spawning temperature but fed 17α MT hormone incorporated feed) (SET 3). The data was collected on 70^th^ day of the experiment.

Fish No.	Replicate 1 BW (g)	Replicate 2 BW (g)	Replicate 3 BW (g)	Fish No.	Replicate 1 BW (g)	Replicate 2 BW (g)	Replicate 3 BW (g)
1	10.4	9.5	9.8	36	8.7	9.5	8.9
2	9	9.5	9.9	37	8.8	9.2	9
3	9.5	9.1	10	38	9	10.2	9.8
4	9.8	8.9	11.2	39	9.7	10.5	8.9
5	10	10.2	10.4	40	9.4	8.4	9.3
6	10	11.4	9.8	41	9.1	8.8	8.9
7	9.6	9.7	9.5	42	8.9	9.1	9.8
8	9.7	9.3	9.6	43	9	9.3	9
9	9.8	9.2	8.9	44	10.6	9.5	8.7
10	9.3	9	9.2	45	9.9	9.7	9.1
11	9	8.7	9.6	46	9.7	9.2	9.8
12	9.1	9.2	9.9	47	9.6	11	8.9
13	8.9	8.6	8.2	48	9.8	10.3	10.9
14	8.7	9.3	8.9	49	10.3	10	8.9
15	8.8	10.3	8.7	50	9.7	9.6	9.3
16	8.6	10.9	9.2	51	9.2	8.7	9.8
17	8.3	11	9.8	52	9.1	8.6	9.2
18	8.2	9.7	9.7	53	10.5	8.5	8.7
19	10	8.3	10.3	54	10.9	8.1	9.1
20	9.3	8.2	10.6	55	9.7	10.9	9.9
21	9.5	9.1	9.5	56	10.9	9.3	8
22	9.7	8.4	9.3	57	8.6	9.2	8.9
23	9.8	8.8	9.5	58	8.8	9.7	9.2
24	8.7	8.2	9.8	59	8.4	10	8.7
25	8.3	8.7	10.2	60	8.2	10	9.4
26	8.2	9.3	8.4	61	8.9	10.5	8.3
27	8.1	10.2	9	62	8	9.8	9.3
28	10	8.3	9.3	63	10.4	8.4	9
29	9.4	8.1	9.4	64	10.8	8.8	8
30	9.3	8.5	9.2	65	8.9	8.7	9.7
31	8.9	9	9.8	66	10	9	8.9
32	9.3	10.3	9.9	67	9.9	9.4	9.8
33	9.4	10.2	9.5	68	9.7	9.1	8.2
34	9.2	9.1	9.9	69	9.5	9.4	8.8
35	8	9.3	10.5	70	9.3	8.9	9

**Table 4a:** Progeny from *O. shiranus* pure crosses in hapas, fed normal National Aquaculture Center formulated fry feed and not incorporated with 17α MT hormone (Control) SET4. The data was collected on the 1^st^ day of the experiment

4: Time Series Data from SET 4.							
Fish No.	Replicate 1 BW (g)	Replicate 2 BW (g)	Replicate 3 BW (g)	Fish No.	Replicate 1 BW (g)	Replicate 2 BW (g)	Replicate 3 BW (g)
1	1.8	2.1	1.9	36	2.2	2.1	1.8
2	1.9	1.9	2	37	2	2.4	2.1
3	2.1	2.3	2.1	38	2.1	2.5	2
4	2	2.1	2.2	39	1.9	1.8	2.1
5	2	2.2	1.8	40	1.7	1.9	1.5
6	2.3	2.5	2.1	41	1.6	2.3	1.8
7	2.2	1.9	1.9	42	1.4	2.1	2
8	2.1	2.1	1.8	43	1.9	2.2	2.2
9	2.1	2.2	1.8	44	1.9	2.2	1.9
10	2	2.1	1.7	45	2.2	2.4	2
11	2	2	2.3	46	2.4	2.1	1.9
12	2	1.9	2.2	47	2.1	2.5	2.1
13	1.8	2.1	2	48	1.4	2.1	2
14	1.9	2.3	2	49	1.8	2.1	1.7
15	2.1	2.1	2.1	50	1.9	2	1.4
16	2	2	2.2	51	2	1.9	1.8
17	2.2	2	1.7	52	1.5	1.8	1.9
18	2.4	1.9	1.6	53	2.1	1.9	2
19	2.1	1.8	1.9	54	2.3	1.7	2.1
20	2	1.5	1.8	55	2	2	2.2
21	2.1	1.8	1.9	56	2	1.9	2
22	1.9	1.7	2.2	57	2.1	1.7	2.1
23	1.9	1.5	1.9	58	2.1	1.4	1.9
24	1.8	1.4	1.9	59	2	2	1.8
25	2.1	2.3	2	60	2.3	1.4	1.6
26	2.3	2.2	2	61	2.1	1.5	2.1
27	2.2	2	2.1	62	2.2	2	1.5
28	2	2	1.2	63	2	2	1.7
29	2.1	2	2.2	64	2	2.1	2
30	1.9	2	2	65	2	2.2	1.9
31	1.8	1.9	2	66	1.9	2	1.4
32	1.9	1.8	1.7	67	1.8	2	1.7
33	2.2	2	1.8	68	2	2	1.9
34	2.1	2.1	2.1	69	1.8	1.9	1.8
35	2.3	2.2	1.9	70	2	1.8	1.3

**Table 4b:** Progeny from *O. shiranus* pure crosses in hapas, fed normal National Aquaculture Center formulated fry feed and not incorporated with 17α MT hormone (Control) SET4. The data was collected on the 14^th^ day of the experiment.

Fish No.	Replicate 1 BW (g)	Replicate 2 BW (g)	Replicate 3 BW (g)	Fish No.	Replicate 1 BW (g)	Replicate 2 BW (g)	Replicate 3 BW (g)
1	2	2.4	2.3	36	1.9	2.1	2.4
2	2.1	2.2	2.5	37	2.3	2.2	2.5
3	2.2	2.2	2.1	38	2	2.4	2.1
4	1.9	2	2	39	2	2.9	2.2
5	2.1	2.3	2	40	2.1	2.4	1.9
6	2.1	2.5	2	41	2	2.2	1.9
7	2	2.7	1	42	2.1	1.9	2.3
8	2.4	2.9	1.7	43	2.1	1.6	2.1
9	2.1	1.9	2.6	44	2	1.6	2.2
10	2.4	1.8	1.7	45	1.9	1.9	2
11	2.3	2.6	1.9	46	2.5	2	2
12	2.1	2.2	1.6	47	1.7	2.1	2
13	2.6	2	2.3	48	2.2	2.9	2
14	2	2	2.1	49	2.1	2.3	2.3
15	1.9	2.1	2.3	50	2.2	2.3	2
16	2.3	2.4	2.2	51	2.3	2.6	2.4
17	2.1	2.7	2.5	52	2.1	2.7	2.5
18	2.6	2.3	2.4	53	2	2.5	2.2
19	2.7	2	2.3	54	2.1	2.6	1.9
20	2	2	2.1	55	2	2	2.3
21	2.1	1.8	2.2	56	2.1	2	2.4
22	2	1.9	1.9	57	2	2	2.8
23	2	2	2.1	58	2.3	2.5	2.5
24	2	2.1	2	59	2	2.6	2.8
25	2.3	2.2	2	60	2.4	2	2.9
26	2	2.4	1.8	61	2	2	2.5
27	2.1	2.4	1.8	62	2.1	1.9	2.6
28	2.2	2.1	1.9	63	2.1	1.8	2.4
29	2.1	2.7	2.2	64	2	1.9	2.1
30	1.9	2.6	2.3	65	2	2.3	2.7
31	2.4	2.3	2.5	66	2.1	2.8	2.9
32	1.9	2.2	2.2	67	2.1	2.9	2.8
33	2	2.1	2	68	2	2.8	2.8
34	2.1	1.8	2.1	69	2.4	2.2	2.9
35	2.3	1.9	2.3	70	1.9	2.3	2.8

**Table 4c:** Progeny from *O. shiranus* pure crosses in hapas, fed normal National Aquaculture Center formulated fry feed and not incorporated with 17α MT hormone (Control) SET4. The data was collected on the 28^th^ day of the experiment

Fish No.	Replicate 1 BW (g)	Replicate 2 BW (g)	Replicate 3 BW (g)	Fish No.	Replicate 1 BW (g)	Replicate 2 BW (g)	Replicate 3 BW (g)
1	2.6	3.2	3.1	36	3.3	3.9	2.7
2	2.9	3.6	3.3	37	3.2	4.1	4
3	3	2.9	3.2	38	3.4	3.2	1.9
4	3.1	1.8	2.9	39	2.9	3.8	1.7
5	3.2	1.9	2.8	40	3.2	3.6	1.9
6	2.2	2.3	2.7	41	3.2	3.9	2.9
7	2.8	3.7	3.1	42	3.1	3.1	2.8
8	2.9	2.9	3.2	43	2.7	3	2.1
9	3.2	3	2.8	44	2.7	3	2.2
10	3.1	3.3	2.7	45	1.8	3.2	3
11	1.9	3.8	3.8	46	2.2	3	3.1
12	2.2	2.9	3.9	47	2	2.9	3.2
13	2.3	4	3.9	48	2	2.8	3.5
14	2.6	1.8	2.9	49	2.6	2.8	3.7
15	3	2.8	3.2	50	2.5	2.5	3.5
16	3.1	3	3.3	51	2.3	2.1	3
17	3.2	3.7	3.1	52	2.6	2.8	3
18	3.8	3.6	3	53	2.7	2.7	3.2
19	2.9	3.1	3.1	54	2.1	2.1	3.6
20	2.1	3.6	3	55	2	2	3.7
21	2.2	3.5	3.6	56	3.2	2	3.6
22	2.8	3.4	3.3	57	3	2.1	2.9
23	2.5	3.9	3.2	58	3.5	2.9	3.1
24	2.7	3.7	3.1	59	2.2	2.1	3.7
25	3.2	3.2	2.9	60	2.4	2	3.8
26	3.1	4.1	2.9	61	2.5	2	3
27	3	4.2	2.3	62	2.3	2	3.4
28	1.8	3.9	2.3	63	2.1	1.9	2
29	2.4	3.8	2.5	64	2.3	1.8	1.9
30	2.3	3.9	3	65	2.6	1.8	1.8
31	2.7	3	3.5	66	3.3	1.7	1.7
32	2.8	3	3.1	67	3.9	1.6	1.5
33	2.9	3.2	3	68	3.8	1.6	2
34	3	3.3	3	69	3.8	3	2.2
35	3.1	3.8	3	70	3.2	2.9	3

**Table 4d:** Progeny from *O. shiranus* pure crosses in hapas, fed normal National Aquaculture Center formulated fry feed and not incorporated with 17α MT hormone (Control) SET4. The data was collected on the 42^nd^ day of the experiment

Fish No.	Replicate 1 BW (g)	Replicate 2 BW (g)	Replicate 3 BW (g)	Fish No.	Replicate 1 BW (g)	Replicate 2 BW (g)	Replicate 3 BW (g)
1	4.7	3.3	4.1	36	3.9	4.8	4.9
2	4.5	3.6	4	37	4.2	3.2	4.8
3	4.3	4.2	3.9	38	4.2	4.2	3.3
4	4.4	3.9	3.6	39	4.1	4.2	3.4
5	4.1	3.9	3.2	40	4.5	4.1	3.5
6	5.5	3.5	3.9	41	4.6	3	3.1
7	5	3.4	4.2	42	4.4	3.4	3.2
8	2.9	3.7	4.3	43	4.9	3.8	3.8
9	3.3	3.8	4.4	44	5	3.4	3.9
10	3.7	3.2	4.6	45	4.2	4.2	4.1
11	3.9	3.1	4.9	46	4.3	3.3	4.2
12	4	4.2	4.8	47	4.8	4.2	3.4
13	4.3	3.3	4.1	48	4.8	3.7	3.2
14	3.6	4.5	3.9	49	4.2	3	3.1
15	3.9	4.7	3.8	50	5.2	2.9	2.9
16	3.6	4.9	4.1	51	4.1	2.8	2.8
17	3.1	5.1	4.3	52	4.2	2.1	2.7
18	3.6	5.2	4.5	53	4.3	2	4.1
19	4.1	5.3	4.7	54	4.9	2	4.5
20	4.5	4.7	4.4	55	5.2	3.1	5
21	4.7	4.9	4.6	56	4	3.2	4.9
22	3.8	4.8	4.8	57	4.2	2.9	4.8
23	4.7	4.7	4.9	58	4.3	2.9	4
24	4.1	4.1	3.3	59	4.7	3.2	3.8
25	4.6	4	3.1	60	4.5	3.3	4.2
26	4.8	3.8	3.6	61	4.2	3.3	4.4
27	4.2	4.3	3.6	62	4.8	4.2	5.1
28	4.2	4.4	3.9	63	5	3.3	4.9
29	4.7	4.2	4.2	64	4.2	3.2	4
30	4.5	3.7	3.1	65	4.2	3.1	4
31	4.6	4.8	3.9	66	5.6	3	4.1
32	4.7	3.3	3.2	67	3.9	3	4.2
33	4.6	3.6	4.5	68	3.1	3.3	4.3
34	4.9	3.3	4.2	69	3.9	3.2	3.9
35	4	3.8	4.7	70	4.3	3.1	3.7

**Table 4e:** Progeny from *O. shiranus* pure crosses in hapas, fed normal National Aquaculture Center formulated fry feed and not incorporated with 17α MT hormone (Control) SET4. The data was collected on the 56^th^ day of the experiment.

Fish No.	Replicate 1 BW (g)	Replicate 2 BW (g)	Replicate 3 BW (g)	Fish No.	Replicate 1 BW (g)	Replicate 2 BW (g)	Replicate 3 BW (g)
1	4.9	5.1	5.2	36	5.6	5	5.1
2	5	5.3	5.6	37	5.4	5.3	4.9
3	5.8	4.8	5.5	38	5.3	5.2	5.2
4	4.4	4.9	5.8	39	5.8	5.9	5.1
5	4.6	4.4	5.3	40	5.9	4	4.9
6	5.6	4.3	6.1	41	5.1	4	5.2
7	5.8	5.6	6	42	5.3	4.3	4.8
8	5.9	5.8	5.4	43	5	5.1	5.3
9	4.1	5.9	5.1	44	5.6	4.8	5.5
10	4.4	4.3	4.8	45	6	4.5	5.7
11	4.7	5.3	4.7	46	6.1	4	5.9
12	4.9	5.4	4.8	47	4.5	4	5.1
13	4.5	5.5	5	48	5.6	3.7	5.6
14	4.7	5.8	4.5	49	5.9	3.9	5.9
15	4.8	5.9	5.6	50	5.4	3.1	5.2
16	5.4	4.9	5.2	51	4.9	4.2	5.7
17	5.3	5.7	4.8	52	5.2	3.3	4.8
18	5.5	5.8	4.7	53	5.1	4.4	4.9
19	5.6	5.3	5.9	54	5.6	4.3	5.2
20	5.8	5.1	6.2	55	6.2	3.7	4.8
21	6.1	5.3	4.8	56	5.4	4.1	5.3
22	5.9	5.9	3.7	57	5.5	4.2	4.8
23	5	4.1	4.8	58	5.9	3.3	4.9
24	5.4	5.4	4.1	59	5.7	3.7	4.2
25	5.3	4.5	4.3	60	5.8	3.8	5
26	5.2	4.3	4.7	61	5.1	4.1	4.1
27	5.4	4.8	5.6	62	5.3	4	5
28	5.3	6.1	4.8	63	5.4	4.5	4.9
29	5.1	6.3	5.1	64	5.3	5.2	4.8
30	4.9	5.9	5.3	65	5.1	5.6	5.7
31	5.4	5.8	4.9	66	6	4.6	5.2
32	5.3	5.7	5.2	67	5.6	4.1	5
33	5.1	5.6	5.1	68	5	4.8	5.9
34	5	5.5	4.8	69	4.6	4.9	4.7
35	5	5.1	4.8	70	4.9	5	4.6

**Table 4f:** Progeny from *O. shiranus* pure crosses in hapas, fed normal National Aquaculture Center formulated fry feed and not incorporated with 17α MT hormone (Control) SET4. The data was collected on the 70^th^ day of the experiment

Fish No.	Replicate 1 BW (g)	Replicate 2 BW (g)	Replicate 3 BW (g)	Fish No.	Replicate 1 BW (g)	Replicate 2 BW (g)	Replicate 3 BW (g)
1	7.8	8.7	8.1	36	7.4	7.4	8.5
2	6.9	8.9	7.9	37	7.2	7.3	8.8
3	7.3	7.8	7.8	38	7.4	7.2	8.9
4	7.8	7.5	7.7	39	8.9	7	7.7
5	7.4	7.1	7.1	40	8.3	8.8	7.9
6	6.5	7.4	6.9	41	8	8.9	7.4
7	6.9	7.3	8	42	8.7	8.1	7.3
8	6.1	7.2	7.9	43	6.1	8.7	8.4
9	6.3	8.9	8.4	44	6.3	8.6	8.3
10	7.9	8.1	8.2	45	6.2	7.4	8.9
11	8	8.5	8.7	46	7.9	7.3	7.4
12	8.3	8.7	7.4	47	8	7.9	7.1
13	8.2	7.9	7.9	48	7.7	7.7	7.9
14	8.1	7.4	8.9	49	7.4	7.8	6.8
15	7.8	7.3	8.1	50	7.2	8.9	6.1
16	7.9	7.1	7.3	51	8	7	6.5
17	7.3	7	7.6	52	7.9	6.9	6.3
18	7.2	6.9	8.8	53	8.2	7.8	6
19	6.9	7.8	8.3	54	8	7	6.1
20	7.1	7.9	8.2	55	8.7	7.1	6.8
21	8.2	8.1	8	56	7.9	6.8	6.3
22	7.8	8.4	8.4	57	7.8	6.8	6.2
23	7.6	8.5	8.6	58	7.1	7.1	6.9
24	7.7	7.6	8.1	59	7.3	6.5	6.1
25	7.2	7.4	8.9	60	7.7	7.1	6.5
26	7.1	7.2	8.6	61	8.9	7	6.6
27	7.6	7.1	8.3	62	6.8	7.8	6.1
28	7.9	8.9	7.2	63	8.5	6.8	6
29	8	8.8	7.6	64	8.2	6.1	7
30	8.3	8.7	8.7	65	8.7	7.9	7.9
31	8.4	8.4	8.5	66	8.8	8	8.1
32	8.1	8.5	8.3	67	8.9	8.3	8.4
33	8	8.6	8.6	68	8.9	8.4	8.9
34	8	7.8	8.9	69	8.2	7.9	8
35	7.9	7.7	8.1	70	8.9	7	7.8

**Table 5:** Data on Sex Ratio determination using Aceto-Carmine method, after 70 days of fish rearing. The Data is presented for each SET, and in replicate within SETs (n=320).

Data on Sex determination on the 70^th^ day using Aceto-carmine method									
No.	BW(g)	Set No.	Replicate No.	Sex	No.	BW (g)	Set No.	Replicate No.	Sex
SET 1									
1	9	1	1	Male	41	10.7	1	2	Male
									
2	10.4	1	1	Male	42	11	1	2	Male
3	8	1	1	Male	43	4.3	1	2	Female
4	11.4	1	1	Male	44	9	1	2	Male
5	7.8	1	1	Male	45	11.8	1	2	Male
6	10.4	1	1	Male	46	5.1	1	2	Male
7	5.6	1	1	Female	47	12	1	2	Male
8	10.2	1	1	Male	48	10.9	1	2	Male
9	11.3	1	1	Male	49	6.5	1	2	Male
10	6	1	1	Male	50	11.8	1	2	Male
11	5.3	1	1	Male	51	7	1	2	Male
12	10.4	1	1	Male	52	11.8	1	2	Male
13	6.1	1	1	Male	53	10.3	1	2	Male
14	10.8	1	1	Male	54	12	1	2	Male
15	11.4	1	1	Male	55	11.3	1	3	Male
16	4.9	1	1	Female	56	6	1	3	Male
17	12.6	1	1	Male	57	10.9	1	3	Male
18	8	1	1	Male	58	10.6	1	3	Male
19	9	1	1	Male	59	11.2	1	3	Male
20	11	1	1	Male	60	12	1	3	Male
21	10.1	1	1	Male	61	11.6	1	3	Male
22	4.3	1	1	Male	62	9	1	3	Female
23	11.6	1	1	Male	63	10.8	1	3	Male
24	11	1	1	Male	64	9	1	3	Male
25	10.6	1	1	Male	65	11.9	1	3	Male
26	4.3	1	1	Female	66	7	1	3	Male
27	10.2	1	1	Male	67	8	1	3	Female
28	12.3	1	2	Male	68	10.8	1	3	Male
29	10.2	1	2	Male	69	11.9	1	3	Male
30	7.6	1	2	Male	70	5.9	1	3	Female
31	11	1	2	Male	71	9.9	1	3	Female
32	10.9	1	2	Male	72	6.2	1	3	Male
33	12	1	2	Male	73	7.2	1	3	Male
34	4.5	1	2	Female	74	6.5	1	3	Male
35	11.9	1	2	Male	75	11.2	1	3	Male
36	12	1	2	Male	76	9.8	1	3	Male
37	11.8	1	2	Male	77	11	1	3	Male
38	6.5	1	2	Female	78	6.8	1	3	Male
39	10.5	1	2	Male	79	6.3	1	3	Male
40	11.8	1	2	Male	80	7	1	3	Male
SET 2									
81	8.7	2	1	Male	121	8.7	2	2	Male
82	8.3	2	1	Male	122	6.2	2	2	Male
83	8.1	2	1	Male	123	9	2	2	Male
84	8	2	1	Male	124	8.9	2	2	Male
85	8	2	1	Male	125	8	2	2	Male
86	7.9	2	1	Male	126	5.5	2	2	Female
87	7.8	2	1	Male	127	6.8	2	2	Male
88	7.7	2	1	Male	128	8.2	2	2	Male
89	7.5	2	1	Male	129	8.1	2	2	Female
90	8	2	1	Male	130	6.2	2	2	Male
91	6.9	2	1	Male	131	4.2	2	2	Male
92	8.2	2	1	Male	132	6.5	2	2	Male
93	8.3	2	1	Male	133	9.2	2	2	Male
94	8.7	2	1	Male	134	6.1	2	2	Male
95	5.1	2	1	Female	135	6.2	2	3	Male
96	8.3	2	1	Male	136	8.9	2	3	Male
97	8.1	2	1	Male	137	8.2	2	3	Male
98	8	2	1	Male	138	5.9	2	3	Male
99	8.7	2	1	Male	139	8.9	2	3	Male
100	8.8	2	1	Male	140	6.1	2	3	Male
101	5.4	2	1	Male	141	6	2	3	Female
102	8.9	2	1	Male	142	4.2	2	3	Female
103	8.2	2	1	Male	143	4.3	2	3	Female
104	8.7	2	1	Male	144	8.8	2	3	Male
105	6.1	2	1	Male	145	8.7	2	3	Male
106	6.1	2	1	Male	146	4.1	2	3	Male
107	8.8	2	1	Male	147	8.3	2	3	Male
108	8.3	2	2	Male	148	8.9	2	3	Male
109	8.2	2	2	Male	149	8.7	2	3	Male
110	8.5	2	2	Male	150	4.5	2	3	Male
111	7.8	2	2	Male	151	8.9	2	3	Male
112	6	2	2	Male	152	6.6	2	3	Male
113	8.6	2	2	Male	153	8.2	2	3	Male
114	8.8	2	2	Male	154	8.8	2	3	Male
115	8.1	2	2	Male	155	8.1	2	3	Male
116	7.2	2	2	Male	156	6.3	2	3	Male
117	8.5	2	2	Male	157	8.8	2	3	Male
118	8.1	2	2	Male	158	4.3	2	3	Male
119	5.5	2	2	Male	159	8.9	2	3	Male
120	9	2	2	Male	160	8.2	2	3	Male
SET 3									
161	7.2	3	1	Male	201	8.7	3	2	Female
162	8.9	3	1	Male	202	8.6	3	2	Male
163	8.7	3	1	Male	203	8.5	3	2	Male
164	8.8	3	1	Male	204	8.1	3	2	Male
165	8.6	3	1	Male	205	9.8	3	2	Male
166	8.3	3	1	Male	206	9.3	3	2	Male
167	8.2	3	1	Male	207	9.2	3	2	Male
168	5.2	3	1	Female	208	9.7	3	2	Male
169	8.1	3	1	Male	209	4.5	3	2	Male
170	6.2	3	1	Male	210	10	3	2	Male
171	6.8	3	1	Male	211	6.7	3	2	Male
172	7.1	3	1	Male	212	5.8	3	2	Male
173	8.7	3	1	Male	213	8.4	3	2	Male
174	8.3	3	1	Male	214	8.8	3	2	Male
175	8.2	3	1	Male	215	8.9	3	3	Male
176	8.1	3	1	Male	216	9.8	3	3	Male
177	6.1	3	1	Female	217	6.2	3	3	Male
178	4.2	3	1	Male	218	8.7	3	3	Male
179	4.1	3	1	Male	219	9.1	3	3	Male
180	7.2	3	1	Male	220	4.2	3	3	Female
181	9.3	3	1	Male	221	8.9	3	3	Male
182	9.4	3	1	Male	222	10.9	3	3	Male
183	6.3	3	1	Male	223	8.9	3	3	Male
184	8	3	1	Male	224	9.3	3	3	Male
185	8.7	3	1	Male	225	9.8	3	3	Male
186	8.8	3	1	Male	226	9.2	3	3	Male
187	6.1	3	1	Male	227	8.7	3	3	Male
188	7	3	2	Male	228	9.1	3	3	Male
189	5.5	3	2	Male	229	6.2	3	3	Male
190	8.4	3	2	Male	230	4.5	3	3	Male
191	4.8	3	2	Male	231	8.9	3	3	Male
192	5.2	3	2	Male	232	9.2	3	3	Male
193	6.1	3	2	Male	233	8.7	3	3	Male
194	7.8	3	2	Male	234	9.4	3	3	Male
195	5.8	3	2	Male	235	8.3	3	3	Male
196	9.2	3	2	Male	236	9.3	3	3	Male
197	4.9	3	2	Male	237	9	3	3	Male
198	5.8	3	2	Male	238	8	3	3	Male
199	10	3	2	Male	239	6.8	3	3	Male
200	9.6	3	2	Male	240	8.9	3	3	Male
SET 4									
241	7.1	4	1	Male	281	7	4	2	Male
242	7.6	4	1	Female	282	7	4	2	Female
243	5.1	4	1	Female	283	6.9	4	2	Female
244	8	4	1	Female	284	7.8	4	2	Male
245	8.3	4	1	Male	285	7	4	2	Male
246	8.4	4	1	Male	286	7.1	4	2	Male
247	8.1	4	1	Male	287	6.8	4	2	Female
248	8	4	1	Male	288	6.8	4	2	Female
249	8	4	1	Male	289	7.1	4	2	Male
250	6.2	4	1	Female	290	6.5	4	2	Female
251	7.4	4	1	Female	291	7.1	4	2	Female
252	7.2	4	1	Female	292	7	4	2	Female
253	7.4	4	1	Female	293	7.8	4	2	Male
254	5.3	4	1	Male	294	6.8	4	2	Female
255	8.3	4	1	Male	295	6.9	4	3	Female
256	8	4	1	Male	296	8	4	3	Male
257	8.7	4	1	Male	297	7.9	4	3	Female
258	6.1	4	1	Female	298	8.4	4	3	Male
259	6.3	4	1	Female	299	8.2	4	3	Male
260	6.2	4	1	Female	300	8.7	4	3	Male
261	7.9	4	1	Male	301	7.4	4	3	Male
262	8	4	1	Male	302	4.1	4	3	Female
263	7.7	4	1	Male	303	5.2	4	3	Female
264	7.4	4	1	Male	304	8.1	4	3	Male
265	7.2	4	1	Female	305	7.3	4	3	Female
266	8	4	1	Male	306	7.6	4	3	Female
267	7.9	4	1	Male	307	7.5	4	3	Male
268	7.3	4	2	Female	308	8.3	4	3	Male
269	7.2	4	2	Female	309	8.2	4	3	Male
270	7	4	2	Female	310	8	4	3	Female
271	8.8	4	2	Male	311	8.4	4	3	Male
272	8.9	4	2	Male	312	8.6	4	3	Male
273	8.1	4	2	Male	313	8.1	4	3	Male
274	8.7	4	2	Male	314	4.3	4	3	Female
275	8.6	4	2	Male	315	8.6	4	3	Female
276	7.4	4	2	Female	216	8.3	4	3	Female
277	7.3	4	2	Female	217	7.2	4	3	Female
278	7.9	4	2	Male	218	3.4	4	3	Female
279	7.7	4	2	Female	219	8.7	4	3	Male
280	7.8	4	2	Female	320	8.5	4	3	Male

SET 1 = Progeny from interspecific mating of *O. karongae* (male) x *O. shiranus* (female); SET 2 = Progeny from *O.shiranus* pure crosses in hapas and fed 17α MT hormone incorporated feed; SET 3 = Progeny from *O. shiranus* pure crosses in re-circulatory hatchery at 27°C (control group for spawning temperature and was also fed 17α MT hormone incorporated feed) and SET 4 = Progeny from *O.shiranus* pure crosses in hapas reared on normal feed without the 17α MT hormone (Control).

Table 1a, Table 2a, Table 3a, Table 4a: Data on Fish Body weights of four SETs of the experiment from initial stocking, i.e., first day of sampling (Tables a), fourteen days after stocking (Tables b), twenty-eight days after stocking (Tables c), forty-two days after stocking (Tables d), fifty-six days after stocking (Tables e) and 70 days after stocking (Tables f) by SET

1: Time Series Data from SET 1 of the Experiment.

## Declaration of Competing Interest

All the authors declare that none of them has known competing financial interests or personal relationships that could have neither potentially influence the work reported in this paper nor publication of this work in the Aquaculture Reports Journal.

## Appendix Supplementary materials

Image, application 1
